# Bridging the immunogenicity of a tetravalent dengue vaccine (TAK-003) from children and adolescents to adults

**DOI:** 10.1038/s41541-023-00670-6

**Published:** 2023-05-25

**Authors:** Inge LeFevre, Lulu Bravo, Nicolas Folschweiller, Eduardo Lopez Medina, Edson Duarte Moreira, Francesco Nordio, Mayuri Sharma, Leslie M. Tharenos, Vianney Tricou, Veerachai Watanaveeradej, Peter J. Winkle, Shibadas Biswal

**Affiliations:** 1 Vaccines Business Unit, Takeda Pharmaceuticals International AG, Zürich, Switzerland; 2 College of Medicine, University of the Philippines, Manila, Philippines; 3 Centro de Estudios en Infectología Pediatrica CEIP; Department of Pediatrics, Universidad Del Valle; Clínica Imbanaco, Grupo Quironsalud, Cali, Colombia; 4 Associação Obras Sociais Irmã Dulce Hospital Santo Antônio and Oswaldo Cruz Foundation, Bahia, Brazil; 5 Takeda Vaccines, Inc., Cambridge, MA USA; 6 The Division of Environmental and Occupational Health Sciences, University of Illinois at Chicago School of Public Health, Chicago, IL USA; 7 Department of Pediatrics, Phramongkutklao Hospital and Faculty of Medicine, Kasetsart University, Bangkok, Thailand; 8 Anaheim Clinical Trials, Anaheim, CA USA

**Keywords:** Viral infection, Vaccines

## Abstract

Immunobridging is an important methodology that can be used to extrapolate vaccine efficacy estimates to populations not evaluated in clinical studies, and that has been successfully used in developing many vaccines. Dengue, caused by a mosquito-transmitted flavivirus endemic to many tropical and subtropical regions, is traditionally thought of as a pediatric disease but is now a global threat to both children and adults. We bridged immunogenicity data from a phase 3 efficacy study of a tetravalent dengue vaccine (TAK-003), performed in children and adolescents living in endemic areas, with an immunogenicity study in adults in non-endemic areas. Neutralizing antibody responses were comparable in both studies following receipt of a two-dose TAK-003 schedule (months 0 and 3). Similar immune responses were observed across exploratory assessments of additional humoral responses. These data support the potential for clinical efficacy of TAK-003 in adults.

## Introduction

While large-scale, placebo-controlled efficacy studies remain the gold standard for evaluating vaccine performance before approval for use in the general population, studies in the entire intended target population are not always achievable. For example, large-scale efficacy trials are challenging to perform, perhaps because the disease is rare overall or in a certain target age group, as the number of participants who would need to be enrolled to allow statistical evaluation of the findings is unfeasibly large^1^. Additionally, factors such as the need for rapid development of vaccines (such as during a pandemic situation) may mean that there is limited time to perform efficacy evaluation in target populations who were excluded from early-phase studies (e.g., children and adolescents)^2^.

Once vaccine efficacy has been established under one set of conditions, immunobridging is a methodology that can be used to infer efficacy in another set of conditions, such as evaluating a different age group, different formulation, or different dosing regimen^3–5^. Immunobridging has been successfully used in the development of a number of vaccines. including 20-valent pneumococcal vaccine^6^, COVID-19^7^, and human papillomavirus (HPV)^8^. For pneumococcal conjugate vaccines (PCVs), and despite the existence of aggregate correlate of protection (CoP), this strategy was used to translate immunogenicity data from a new-generation vaccine with increased valence, from a different age group, or from a vaccine made by a different manufacturer. Likelihood of protection against pneumococcal diseases and nasopharyngeal carriage based on immunobridging to an early PCV, which was initially licensed in infants and toddlers on basis of efficacy trial data^9–11^, has been accepted by national regulatory authorities^12,13^, especially when there was the commitment to generate direct evidence of effectiveness in post-licensure studies^14,15^. The same strategy was applied for a new-generation COVID-19 vaccine when conducting efficacy trials became unethical or impractical^7^. For the HPV vaccines, immunobridging was used to support their use in girls aged 9–15 years, assuming that efficacy was comparable if the antibody responses were non-inferior in young women aged 16–26 years, among whom clinical efficacy against a surrogate endpoint for cervical cancer was demonstrated^16,17^. Immunobridging based on demonstrating non-inferiority of the immune responses was also used to support the further extension of the indications to include use in women aged 27–45 years and in boys and men aged from 9–47 years (supported by demonstrated efficacy against genital warts and a surrogate endpoint for anal cancer in men aged 16–26 years), to facilitate clinical development of a 9-valent HPV vaccine, and to support a dose-schedule reduction (from 3 to 2 doses)^18–23^.

While half of the world’s population is estimated to live in areas at risk of infection with dengue viruses (DENVs), the majority of cases occur in children and adolescents^24^. However, in some areas, such as Thailand, the mean age of DENV infection is increasing, with approximately 30–40% of cases occurring in adults^25^. Additionally, dengue poses a significant risk to international travelers, accounting for more febrile illness cases in returning travelers from South-East Asia than malaria^26^. While traditionally considered a pediatric disease, adults experience a substantial burden of dengue, meaning that evaluation of potential vaccine performance in this population remains important. Furthermore, comorbidities such as diabetes, cardiovascular disease, renal impairment, and respiratory disease, which are much more prevalent in adults than children, have been identified as significant risk factors for developing severe dengue^27,28^. In dengue-endemic areas, a significant proportion of the population has experienced at least one DENV infection early in life. This limited number of dengue-naïve individuals, which is arguably the most important population from the point of a dengue vaccine development, precludes conducting an efficacy study in adults living in endemic areas^29^, and the lack of CoP means that antibody titers cannot be directly translated in an estimate of protection from dengue. In addition, infection with DENV results in type-specific and both transient and longer-lasting heterotypic antibodies^30^, presenting additional challenges both for evaluating dengue vaccine performance outside of an efficacy study and establishing a CoP. Immunobridging between children/adolescents and adult populations has been performed for the only currently licensed tetravalent dengue vaccine, CYD-TDV, with expected vaccine efficacy higher in adults and in recipients who had previously been exposed to DENV^31,32^.

Given the limitations of robustly assessing dengue vaccine efficacy in adults, we performed immunobridging between immunogenicity data obtained in the large-scale, placebo-controlled efficacy study of the tetravalent dengue vaccine TAK-003, performed in children and adolescents aged 4–16 years living in dengue-endemic regions of Asia and Latin America (DEN-301; NCT02747927), and a phase 3 study in adults aged 18–60 years living in regions of the United States considered non-endemic for dengue (DEN-304; NCT03423173). To minimize the potential for confounding factors, we restricted the analysis to participants who were dengue seronegative at baseline (i.e., the reciprocal titer of dengue-neutralizing antibodies < 10 for all four serotypes).

## Results

### Demographics and baseline characteristics

Overall, 702 baseline seronegative children and adolescents (aged 4–16 years) and 379 baseline seronegative adults (aged 18–60 years) were included in this analysis. Across the two studies, 50–53% of participants were female. Ethnicity was not recorded for the DEN-301 study, but race varied between the two studies, with the majority of participants in DEN-301 being Asian or American Indian/Alaska Native (85%) compared with White (79%) in DEN-304 (Table 1).

**Table 1:** Demographic and baseline characteristics of seronegative participants included in the immunobridging analysis.

	Age 4–16 years DEN-301 (*N* = 702)	Age 18–60 years DEN-304 (*N* = 379)
Age, mean (SD), years	8.5 (3.06)	41.2 (12.29)
Sex, n (%)		
Male	348 (49.6)	177 (46.7)
Female	354 (50.4)	202 (53.3)
Ethnicity, n (%)		
Hispanic or Latino	0 (0.0)	25 (6.6)
Not Hispanic or Latino	0 (0.0)	354 (93.4)
Unknown	702 (100.0)	0 (0.0)
Race		
American Indian or Alaska Native	296 (42.2)	2 (0.5)
Asian	300 (42.7)	2 (0.5)
Black or African American	61 (8.7)	71 (18.7)
Native Hawaiian/Pacific Island	0 (0.0)	1 (0.3)
White	22 (3.1)	299 (78.9)
Multiracial	23 (3.3)	4 (1.1)
Height, mean (SD), cm	129.4 (18.4)	171.8 (10.2)
Weight, mean (SD), kg	30.5 (14.0)	82.6 (15.7)
BMI, mean (SD), kg/m^2^	17.3 (3.6)	27.9 (4.3)

*BMI* body mass index, *SD* standard deviation.

### Comparison of geometric mean titers (GMTs) of dengue-neutralizing antibodies

To compare the immunogenicity of TAK-003 in recipients aged 4–16 years and those aged 18–60 years, GMTs of dengue-neutralizing antibodies assessed via microneutralization assay (MNT_50_; expressed as the reciprocal of the dilution of test serum that shows a 50% reduction in plaque counts compared with that of virus controls) were calculated for each age group. Non-inferiority of GMTs was concluded for individual serotypes if the upper bound of the 95% confidence interval (CI) for the geometric mean ratio (GMR) between the two age groups was < 2.0.

**Fig. 1:**
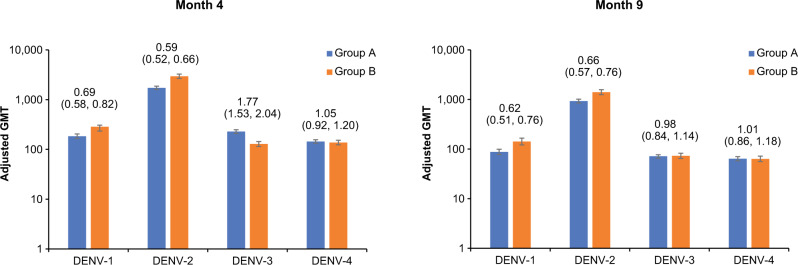
GMRs of dengue-neutralizing antibodies. Adjusted GMRs (95% CI) of dengue-neutralizing antibodies reported for each serotype in baseline seronegative participants aged 4–16 years (DEN-301; Group A) vs. aged 18–60 years (DEN-304; Group B) (per protocol sets)^33^. Abbreviations: CI confidence interval, DENV dengue virus, GMR geometric mean ratio, GMT geometric mean titer.

As previously described in brief in Rivera et al. ^33^, the highest GMTs in both age groups were observed against DENV-2, with lower GMTs against the other three serotypes (Fig. 1). At month 4, non-inferiority of immunogenicity was concluded for DENV-1, DENV-2, and DENV-4, with the adult age group having lower GMTs against DENV-3 than the younger age group at this time point (128.9 vs. 228.0, respectively). By month 9, non-inferiority could be concluded for all four serotypes, with the highest GMTs against DENV-2, followed by DENV-1, DENV-3, and DENV-4.

**Fig. 2:**
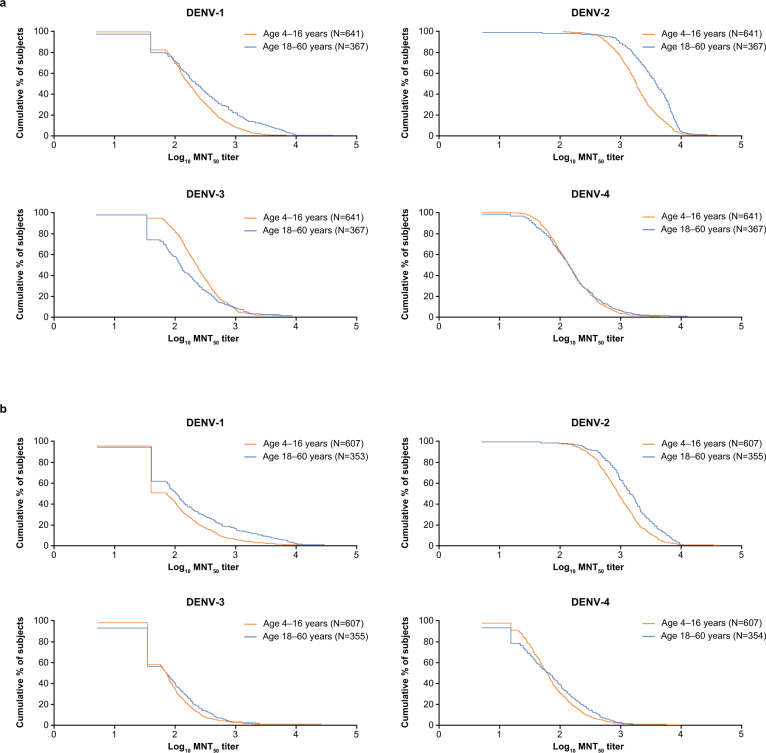
RCDCs of dengue-neutralizing antibodies. RCDCs of dengue-neutralizing antibodies reported for each serotype at (**a**) month 4, and (**b**) month 9 in baseline seronegative participants aged 4–16 years (DEN-301) vs. 18–60 years (DEN-304) (per protocol sets). Abbreviations: DENV dengue virus, MNT_50_ microneutralization titer assay resulting in 50% reduction in plaque counts compared with that of virus controls, RCDC reverse cumulative distribution curve.

Reverse cumulative distribution curves (RCDCs) of log_10_ MNT_50_ titer showed that, at month 4, a slightly higher proportion of adults had higher titers against DENV-1 and DENV-2 than children and adolescents in the DEN-301 study (Fig. 2). This trend was reversed for DENV-3, and for DENV-4 the RCDCs were similar for both age groups. By month 9, the same trends were still observed for DENV-1 and DENV-2, although less pronounced, and the titer distributions against DENV-3 and DENV-4 were the same for both age groups.

**Fig. 3:**
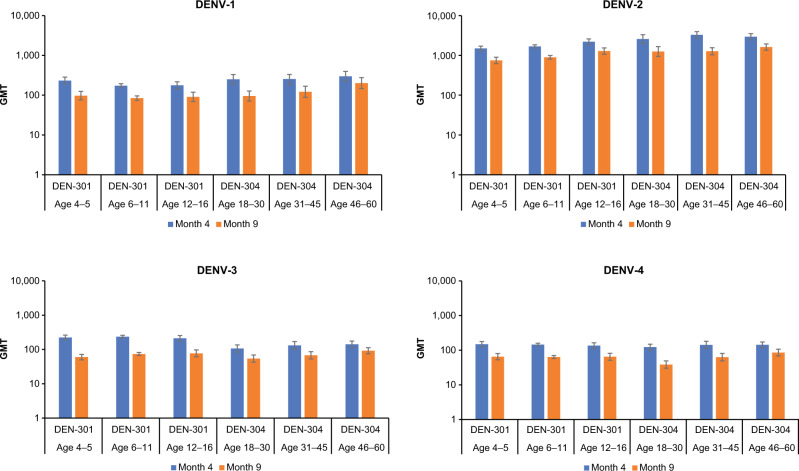
GMTs of MNT_50_ dengue-neutralizing antibodies. GMTs (95% CI) of MNT_50_ dengue-neutralizing antibodies reported for each serotype by age group in baseline seronegative participants (per protocol sets). Abbreviations: CI confidence interval, DENV dengue virus, GMT geometric mean titer, MNT_50_ microneutralization titer assay resulting in 50% reduction in plaque counts compared with that of virus controls.

**Fig. 4:**
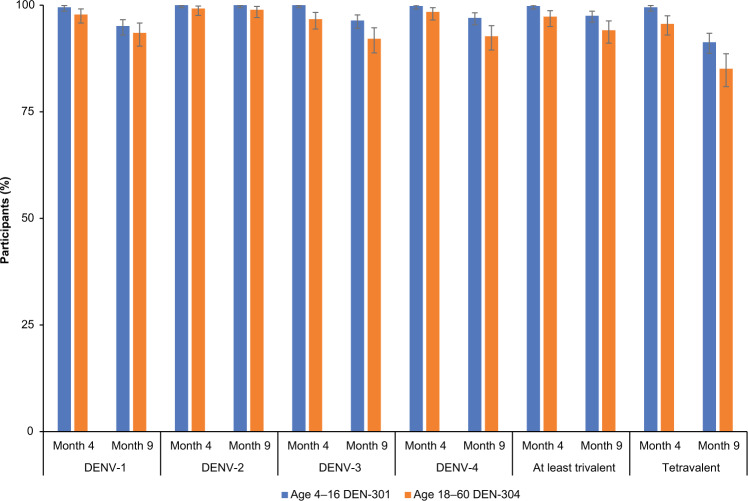
Seropositivity rates for each dengue serotype. Seropositivity rates (95% CI) reported for each dengue serotype in baseline seronegative participants aged 4–16 years (DEN-301) and aged 18–60 years (DEN-304). Abbreviations: CI confidence interval, DENV dengue virus.

No clear differences or trends in GMTs were observed with increasing age within either study population (Fig. 3). Seropositivity rates (i.e., the proportion of participants with post-vaccination reciprocal MNT_50_ titers ≥ 10) were high for each age group at months 4 and 9, with seropositivity rates against individual serotypes ranging from 92% to 100% (Fig. 4).

### Characterization of the immune response

A number of investigations of humoral immune responses to TAK-003 were performed for a subset of participants in each study (Table 2). Effective binding antibodies can prevent flavivirus infection by blocking virus attachment to the cell surface or virus neutralization, by interfering with membrane fusion, or via functional antiviral activities such as activation of Fc-dependent effector functions, including complement activation^34^. TAK-003 assessment showed comparable total binding antibody levels directed against the whole virion between the age groups. Anti-viral binding antibodies constitute neutralizing antibodies and those with effector functions. TAK-003 elicits tetravalent neutralizing antibody responses in both the pediatric and adult population, with highest titers against DENV-2^35–37^.

**Table 2:** Exploratory immunology assessments and readouts for each serotype.

Measure	Time point	Age group	DENV-1	DENV-2	DENV-3	DENV-4
**Anti-dengue NS1 IgG concentration (EU/mL)**	Month 4	4–16 years	245.7 (213.0, 283.6)	1,053.0 (882.2, 1,256.8)	219.2 (189.8, 253.3)	178.7 (149.5, 213.7)
		18–60 years	299.2 (232.8, 384.4)	1,299.9 (1,032.1, 1,637.1)	210.3 (157.8, 280.4)	144.1 (106.0, 196.0)
	Month 9	4–16 years	140.8 (118.3, 167.7)	365.5 (303.4, 440.2)	101.4 (88.3, 116.4)	85.8 (73.0, 100.8)
		18–60 years	185.3 (146.6, 234.2)	624.5 (494.3, 789.0)	114.3 (87.6, 149.2)	83.8 (64.1, 109.7)
**Dengue total binding IgG concentrations (RU/mL)**	Month 4	4–16 years	1,165.8 (937.6, 1,449.6)	1,292.0 (1,060.7, 1,573.7)	1,414.4 (1,180.2, 1,695.1)	641.3 (528.5, 778.1)
		18–60 years	1,056.9 (826.3, 1,352.0)	1,263.5 (1,005.8, 1,587.1)	951.3 (774.4, 1,168.5)	469.9 (370.5, 596.0)
	Month 9	4–16 years	735.2 (579.8, 932.2)	894.3 (710.2, 1,126.1)	835.4 (670.6, 1,040.8)	465.4 (370.9, 583.9)
		18–60 years	723.6 (544.7, 961.3)	844.3 (653.2, 1,091.1)	610.5 (486.4, 766.2)	329.5 (257.3, 421.9)
**Anti-dengue complement antibody titers (EU/mL)**	Month 4	4–16 years	36.8 (27.9, 48.7)	31.6 (24.4, 40.8)	33.6 (25.7, 43.9)	12.7 (9.5, 16.9)
		18–60 years	25.3 (18.8, 34.0)	24.5 (19.0, 31.7)	19.3 (14.3, 26.1)	9.6 (7.3, 12.6)
	Month 9	4–16 years	17.1 (11.6, 25.0)	15.3 (10.8, 21.6)	13.3 (9.0, 19.8)	6.4 (4.5, 8.9)
		18–60 years	14.7 (10.4, 20.8)	13.1 (9.5, 18.1)	9.6 (6.8, 13.6)	5.9 (4.3, 8.0)
Anti-dengue IgG avidity (nm*s)	Month 4	4–16 years	526.7 (300.4, 923.6)	424.2 (225.8, 796.7)	307.2 (161.5, 584.6)	147.3 (70.4, 307.8)
		18–60 years	796.7 (433.3, 1,464.9)	813.4 (466.5, 1,418.1)	403.8 (217.5, 749.8)	224.2 (105.4, 477.0)
	Month 9	4–16 years	222.8 (98.6, 503.8)	315.0 (146.2, 679.0)	92.8 (37.9, 227.3)	35.1 (13.6, 91.1)
		18–60 years	829.3 (477.7, 1,439.6)	931.7 (629.8, 1,378.4)	330.5 (163.7, 667.4)	108.8 (42.6, 278.0)
**Percent of type-specific neutralizing antibody response/EC**_**50**_ **(RVP) after DENV-2 nAb depletion (%)**	Month 4	4–16 years	47.4 (31.0, 64.2)	DeMaso et al. ^38^	57.1 (39.4, 73.7)	94.4 (81.3, 99.3)
		18–60 years	68.0 (46.5, 85.1)	DeMaso et al. ^38^	72.2 (46.5, 90.3)	91.3 (72.0, 98.9)
	Month 9	4–16 years	20.0 (5.7, 43.7)	DeMaso et al. ^38^	47.1 (23.0, 72.2)	100.0 (83.2, 100.0)
		18–60 years	76.2 (52.8, 91.8)	DeMaso et al. ^38^	37.5 (15.2, 64.6)	94.4 (72.7, 99.9)

*CI* confidence interval, *DENV* dengue virus, *EC50* 50% effective concentration, *EU/mL* effective units per milliliter, *IgG* immunoglobulin G, *nAb* neutralizing antibody, *NS1* nonstructural protein 1, *RU/mL* relative units per milliliter, *RVP* reporter virus particle.

Summary of exploratory immunology assessments and readouts for each serotype in participants aged 4–16 years (DEN-301) vs. aged 18–60 years (DEN-304). Data are presented as geometric means (95% CIs) unless otherwise stated. A total of 48 participants were included in the analysis in each study.

Evaluation of the proportion of type-specific neutralizing antibodies after DENV-2 depletion showed that TAK-003 elicits both type-specific and cross-reactive antibodies against DENV-1, DENV-3, and DENV-4 in both age groups^38^. Additionally, complement-fixing antibodies were elicited against all four serotypes, and titers were of similar magnitudes across serotypes and age groups. In a separate study, antibodies produced following vaccination with TAK-003 were also functional in both activating the complement system and neutralizing infection by all DENV serotypes^39^.

Evaluation of anti-dengue immunoglobulin G (IgG) avidity, a measure of the binding strength of induced antibodies, demonstrated that the tetravalent binding antibodies elicited by TAK-003 were affinity-matured in both age groups. This polyclonal antibody maturation optimizes antibody affinity, resulting in increased neutralizing response^40^.

Finally, TAK-003 also elicited anti-non-structural protein 1 (anti-NS1) antibody responses against the DENV-2 NS1 of the vaccine backbone, which was cross-reactive against NS1 from the other DENV serotypes, with similar magnitudes of responses across the two age groups. NS1 is a non-structural protein essential for viral replication and a viral toxin that can contribute to vascular leakage by initiating a cascade of endothelial layer hyperpermeabilization^41–43^. NS1-specific antibodies induced by TAK-003 have been shown to block NS1-mediated endothelial hyper-permeabilization in vitro^41,44^.

## Discussion

We demonstrate that neutralizing antibody responses are comparable in pediatric and adult vaccine recipients. The RCDCs of dengue-neutralizing antibodies and seropositivity rates were similar, with over 95% of individuals achieving tetravalent seropositivity one month following vaccination, regardless of age group. Additionally, we observed comparable levels of antibody responses in various exploratory immunogenicity assessments. These findings suggest that the protective effect of TAK-003 in the adult population can be inferred from the clinical efficacy profile of TAK-003 from the pediatric population studied in the pivotal efficacy study (DEN-301).

The scientific principle of the applied analysis was to demonstrate similar levels of immune response elicited in both trial populations and thereby to infer similar levels of expected protection. TAK-003 elicits tetravalent neutralizing antibodies, which are important in protection against flavivirus infections and are generally regarded as the most relevant marker for protective immunity against DENV. Certain levels of neutralizing antibodies to yellow fever, Japanese encephalitis, or tick-borne encephalitis are widely accepted to correlate with protection^45–47^. Neutralizing antibodies to dengue are also associated with a reduced risk of infection or severe disease caused by DENV^48^.

The determination of DENV antibody CoPs is complicated by serological cross-reactivity among the four dengue serotypes. It is possible that CoP for each of the four serotypes could be different from one another. To date, a definite immunological CoP has not been established. However, analyses of neutralizing antibody responses elicited by TAK-003 indicate an association between a higher magnitude of neutralizing antibody titers and a lower risk of dengue^33^.

While the neutralizing antibodies measured by MNT serve as a reproducible measure of immune response to TAK-003 for comparison purposes, multiple components of the immune system likely contribute to protection elicited by vaccination with TAK-003. For instance, studies of immunity to the YF-17D vaccine, arguably one of the most efficacious vaccines, have demonstrated a broad range of immune responses elicited after vaccination that may contribute to protective immunity^49^. Furthermore, immune mechanisms that prevent infection are not necessarily the same as the mechanisms that clear viral infections or restrict pathogenesis. While many viral vaccines block infection by eliciting functional antibodies, viral clearance, and recovery can also be mediated by cellular immune mechanisms^50^. Even after serum antibody levels decline, memory B cells and T cells are maintained in the immune repertoire. Upon encountering the same pathogen, memory immune cells are reactivated to produce rapid and powerful recall responses^51,52^. Thus, a broad range of immune responses encompassing multiple arms of the immune system may contribute to the prevention and clearance of viral infections. We broadly characterized the immune responses elicited by TAK-003 in the context of previously established principles of antiviral immunity, flavivirus immunity, and vaccine correlates of protection^53^, including neutralizing antibodies (type-specific and cross-reactive), binding antibodies, complement-fixing antibodies, polyclonal antibody avidity, and antibody response to NS1. TAK-003 elicited comparable levels of antibody responses across a wide age range of vaccine recipients across the two phase 3 studies.

Dengue exposure increases with age in endemic areas and is known to positively influence subsequent immune response, both in magnitude and quality. The use of baseline seronegative participants for this analysis was considered appropriate because it ensures that the two populations are comparable, except for the age factor. In dengue-endemic countries, there are practical constraints in screening and enrolling seronegative adult participants. Similarly, enrolling children in non-dengue-endemic countries in a study using an experimental vaccine presents practical difficulties. To address the above-mentioned challenges, it was determined that a comparison of the TAK-003 arms from two separate trials would be a reasonable approach. Additionally, we assumed that vaccine efficacy in the baseline seropositive population will at least be like that in baseline seronegative participants. This is supported by the efficacy data in the pivotal trial and is therefore realistic. Besides, the seropositive group is immunologically heterogeneous due to the type and number of past dengue exposures. This limits any meaningful comparison on immunobridging studies in that population.

The World Health Organization (WHO) guidance on dengue vaccine development recommends careful evaluation of the scientific arguments for and against extrapolation of the efficacy results between populations that differ in a specific characteristic. In that context, this analysis did not suggest any evidence of lower immune response in adults than in children. Exploratory subgroup analysis in the pivotal efficacy trial has also not indicated any evidence of potentially lower efficacy with higher age^33^.

Throughout the clinical development program, we have noted considerable inter-participant variability in the immune response to TAK-003 measured in the neutralizing assay. Therefore, we selected a non-inferiority margin of 0.5/2.0 for statistical comparison, as used to assess lot-to-lot consistency in the DEN-304 study.

In the context of dengue vaccine development, there is an emphasis on measuring antibody response away from the time of vaccination. This is relevant in the context of TAK-003, which has a DENV-2 backbone and so elicits a dominant immunological response, implying the possibility of cross-reactivity. While month 4 was chosen to capture the peak of the immunological response to TAK-003 in accordance with a typical time point for immunogenicity assessment in the clinical development program, we also assessed immunobridging at month 9. It was reassuring to see that immune responses at that time point were statistically comparable, indicating similar longer-term immunogenicity, overall.

We would like to highlight some of the limitations of this analysis. Immunobridging analysis was not intended at the time each trial was designed, hence data were not collected concurrently. Additionally, no sample size calculation was performed, and all eligible participants in the respective studies were included. We did not present any data on cell-mediated responses because they were not investigated in either of these two trials, but have been evaluated in other trials in the clinical development program. Nonetheless, we believe that none of the above undermine the analysis’s conclusions.

While the peak of dengue transmission varies by country and endemicity, dengue is a significant health problem for adults in all dengue-endemic countries, as well as adult travelers to these locations. It is likely that dengue control will require a multimodal approach, with vaccination playing a significant role at some stage. Additionally, large-scale community immunization initiatives will be required to have a substantial impact, and they must consider all affected age groups.

In summary, immunobridging can be regarded as a reasonable alternative to practically difficult placebo-controlled efficacy trials in dengue-naïve adults in the context of the development of a dengue vaccine. The present analysis shows biologically comparable TAK-003 immune response in seronegative adults as in the pediatric population and may therefore be used to infer comparable vaccine protective effects in both adult and pediatric populations.

## Methods

### Studies included

DEN-301 was a large-scale efficacy study performed in healthy children aged 4–16 years living in regions of Asia and Latin America considered endemic for dengue (NCT02747927). Participants were randomized 2:1, stratified by age category and region, to receive a two-dose schedule of TAK-003 or placebo, administered 3 months apart. Full details of the study design and inclusion criteria have been published previously^54^. The study is currently ongoing and has recently completed the fourth year of safety and efficacy follow-up.

DEN-304 was designed as a lot-to-lot consistency study in adults aged 18–60 years living in parts of the United States considered non-endemic for dengue (NCT03423173). Participants were randomized 1:1:1:1 to receive one of three lots of the two-dose TAK-003 formulation, or placebo, administered 3 months apart.

Both trials were conducted in accordance with the Declaration of Helsinki and the International Council for Harmonisation Tripartite Guidelines for Good Clinical Practice, as well as in accordance with applicable local regulations. Informed assent or consent forms and the trial protocol and its amendments were reviewed and approved by institutional review boards (IRBs), independent ethics committees (IECs), and health authorities. Details of IRBs and IECs are outlined in Table S1. Written informed assent or consent was obtained from all participants (or their parents or legal guardians) before enrollment.

### Immunogenicity measures

Immunogenicity was assessed in terms of GMTs of dengue-neutralizing antibodies, as measured by a dengue microneutralization titer assay resulting in ≥ 50% reduction in titer (MNT_50_). The validated MNT_50_ assay used in these studies is in accordance with the WHO guidance on the evaluation of dengue vaccine immunogenicity^55^. The immunogenicity of the vaccine was also assessed in terms of seropositivity to each serotype, with seropositive being defined as a reciprocal MNT_50_ titer ≥ 10. Pre-existing seropositivity was evaluated at baseline, with only participants who were seronegative (i.e., MNT_50_ < 10 for all serotypes) included in this analysis. Only seronegative TAK-003 participants were included to standardize the population across regions and because it was assumed, based on evidence from the clinical development program, that vaccine efficacy was at least as high in baseline seropositive as seronegative participants^33,36,54,56^. For the current analysis, as previously presented briefly in Rivera et al. ^33^, comparisons of GMTs and seropositivity between participants in the two studies were performed at months 4 and 9 (i.e., one and six months after receipt of the second dose of TAK-003).

### Exploratory immunology analysis

Exploratory immunology analyses were performed on a subset of 48 seronegative participants from each study. This subset was selected from participants who consented for their samples to be used for future research and was based on the availability of a sufficient volume (≥500 µL) of serum from both pre- and postvaccination time points. Participants with virologically confirmed or asymptomatic dengue were excluded if they were positive for polymerase chain reaction or non-NS1 positive, or showed a four-fold rise in MNT titers for any serotype. For the DENV-2 and reporter virus particle (RVP) assay to assess type-specific neutralizing antibody response, samples were further selected with a minimum tetravalent MNT_50_ of 20 being required for inclusion.

To understand the immune response profile in seronegative adults, representative of travelers from non-endemic areas, we characterized the spectrum of immune responses elicited by TAK-003 using samples from DEN-304 collected on days 1, 120, and 270 from 48 baseline seronegative participants, who were randomly selected from the group vaccinated with TAK-003 Lot 3 (Group 3), using a number of non-GxP exploratory assessments. The analysis population for the primary immunobridging evaluation was restricted to baseline seronegative participants to minimize the influence of confounding factors.

Full methodologies for all the exploratory immunology assessments have been published previously. Geometric mean concentrations of anti-DENV NS1 were evaluated using the methods previously described in full in Sharma et al., 2020^41^. Briefly, 96-well microtiter plates were coated with DENV NS1 (1.0 μg/mL) in carbonate/bicarbonate buffer pH 9.6 overnight at 4 °C. Following washing (phosphate buffered saline-tween (PBS-T)) and blocking (SuperBlock T20 PBS Blocking Buffer) steps, serum samples were added, and the plates incubated with horseradish peroxidase-conjugated goat anti-human IgG-gamma chain. After washing, color was developed with ABTS Peroxidase Substrate, stopped with 1×ABTS Peroxidase Stop Solution, and optical density at 405 nm measured using a Spectramax 384 Plus plate reader. The concentration of anti-DENV NS1 IgG in serum samples was determined relative to the reference standard and expressed in relative units/mL (RU/mL).

Antibody avidity was measured using Octet HTX systems, as described in Tsuji et al., 2021^40^. Briefly, anti-dengue polyclonal IgG (125 μg/mL) in 0.1% bovine serum albumin (BSA)-PBST were purified from serum by Protein G Sepharose and bound to the biotinylated Viral-like particle (VLP)-captured high-precision streptavidin biosensor for 1800 seconds. The sensor was then incubated with dissociation buffer (0.1% BSA-PBST, 0.35 M NaCl) for 1200 seconds.

Anti-dengue complement-fixing antibody titers were assessed using the Luminex assay, as described in full in Nascimento et al., 2021^57^. Briefly, VLP-coupled microspheres at 25 microspheres/µL were incubated with serum samples for 1 hour and washed with PBS-T, 50 µL/well of plasma-derived purified human C1q at 4 µg/mL added and then incubated for 30 minutes. Following PBS-T washes, 50 µL/well of a polyclonal sheep IgG anti-human C1q at 6.4 µg/mL was added and incubated for 30 minutes. After two additional PBS-T washes, the microspheres were incubated with 50 µL/well of an anti-sheep IgG conjugated to phycoerythrin at 10 µg/mL for 30 minutes. The microspheres were washed with PBS-T, reconstituted with assay buffer, and read on a Luminex plate reader (Magpix or FlexMap 3D). Anti-dengue virus complement-fixing antibody concentrations were calculated relative to a reference standard.

Dengue total binding IgG concentrations were assessed using a dengue-specific sandwich (capture) IgG ELISA previously used in Michlmayr et al., 2021^58^. Briefly, 96-well microtiter plates were coated with pan-flavivirus 4G2 monoclonal antibody (1.0 μg/mL) in carbonate/bicarbonate buffer pH 9.6 overnight at 4 °C. Following washing with PBS-T and blocking (SuperBlock T20 PBS Blocking Buffer) steps, monovalent dengue vaccine drug substance was added, followed by serum samples, and the plates were incubated with horseradish peroxidase-conjugated goat anti-human IgG-gamma chain. After washing, color was developed with ABTS Peroxidase Substrate, stopped with 1×ABTS Peroxidase Stop Solution, and optical density at 405 nm measured using a Spectramax 384 Plus plate reader. The concentration of anti-dengue binding antibodies in serum samples was determined relative to the reference standard and expressed in relative units/mL (RU/mL).

As studies have shown that TAK-003 induces higher neutralizing antibody titers against DENV-2 than the other serotypes^33,36,54,56^, together with the presence of DENV-2 type-specific neutralizing antibodies in post-vaccination samples^59^, type-specific neutralizing antibody responses against DENV-1, -3, and -4 were evaluated using DENV-2 depletion and RVP assay, as described in DeMaso et al. ^38^. Briefly, Tosyl-activated Dynabeads coupled with pan-flavivirus 4G2 monoclonal antibody were bound with either live DENV-2 or BSA control, blocked with BSA, and incubated with heat-inactivated vaccine-recipient sera to deplete DENV-2 targeting antibodies. Depletion was repeated 6 times and remaining neutralizing antibodies were quantified using an RVP neutralization assay. The RVPs were incubated with serial dilutions of DENV-2-depleted vaccine-recipient sera. Raji DC-SIGN cells were infected with these immune complexes and the luciferase readout measured 72 hours post infection. Results were reported as half-maximal effective concentration (EC_50_), the reciprocal dilution of serum required to neutralize 50% of the input RVP, for each serotype after mock or DENV-2 depletion.

### Statistical analysis

Differences in GMTs for each of the four dengue serotypes between the 4- to 16-years age group (DEN-301; Group A) and the 18- to 60-years group (DEN-304; Group B) were estimated using an analysis of (co)variance model with natural logarithms of titers as a response variable and covariates including natural logarithm of baseline titer, and interaction terms. Mean titers, mean differences, and 95% CIs for the difference were anti-log-transformed to obtain group GMTs, between-group GMRs, and 95% CIs for the ratio.

For the primary post-hoc comparison based on GMT data at month 4 (one-month postsecond vaccine dose), non-inferiority was concluded for a given serotype if the upper bound of the respective 95% CI for the GMR between the two studies was below 2.0. Overall noninferiority of immune response was formally concluded if non-inferiority was shown for all four serotypes, therefore no multiplicity adjustment was applied.

No statistical hypotheses were tested in the exploratory immunology analysis; data are presented as GMTs or geometric mean concentrations, as appropriate, plus corresponding 95% CIs. Ninety-five percent CIs were calculated by the Clopper-Pearson method for serotype specificity and using t-distribution critical values for avidity measures. All analysis was performed using SAS version 9.2 or higher.

### Reporting summary

Further information on research design is available in the Nature Research Reporting Summary linked to this article.

## Supplementary information

Table S1. IRB and IEC Information for the DEN-301 and DEN-304 trials

REPORTING SUMMARY

## Data Availability

The datasets, including the redacted study protocol, redacted statistical analysis plan, and individual participants data supporting the results of the completed studies, will be made available within three months from initial request, to researchers who provide a methodologically sound proposal. The data will be provided after its de-identification, in compliance with applicable privacy laws, data protection and requirements for consent and anonymization. Data requests should follow the process described in the Data Sharing section on https://clinicaltrials.takeda.com/ and https://vivli.org/ourmember/takeda/.
