# Supplementary material for: Bridging the immunogenicity of a tetravalent dengue vaccine (TAK-003) from children and adolescents to adults
Source: NPJ Vaccines. 2023 May 25;8:75. doi: 10.1038/s41541-023-00670-6 (PMC10208910; doi:10.1038/s41541-023-00670-6)
Supplement: Supplementary file 1 — Table S1. IRB and IEC Information for the DEN-301 and DEN-304 trials [file 41541_2023_670_MOESM1_ESM.pdf]

Central IRB/IECs	Comissao Nacional de Etica em Pesquisa (CONEP) -Unidade II do Ministerio da Saude<br>SEPN 510-Norte -Bloco A-1º Subsolo- Edificio<br>Brazil
	Comité de Ética en la Investigación CAIMED<br>Carrera 42 No. 17-50<br>Santa Fe de Bogotá D.C. Cundinamarca 111611<br>Colombia
	Ethics Review Committee<br>Faculty of Medical Sciences, University of Sri Jayawardenepura,<br>Gangodawila, Nugegoda, 10250<br>Sri Lanka
Local IRB/IECs	Comitê de Ética em Pesquisa do Hospital Universitário Cassiano Antônio de Moraes - CEP/HUCAM<br>Avenida Marechal Campos, 1355-Santos Dumond<br>Vitória, Espírito Santo 29040-001<br>Brazil
	Comitê de Ética em Pesquisa do Hospital Santo Antônio / Obras Sociais Irmã Dulce<br>Av. Bonfim, 161 Largo de Roma<br>Salvador BA 40415-000<br>Brazil
	Comitê de Ética em Pesquisa em Seres Humanos da Universidade Federal de Mato Grosso do Sul-UFMS<br>Cidade Universitária -Caixa Postal 549 Pró-Reitoria de Pesquisa e Pós-Graduação-PROPP<br>Campo Grande MS 79070-900<br>Brazil
	Comitê de Ética em Pesquisa da Liga Norte Riograndense Contra o Câncer<br>Rua Dr Mário Negócio, 2267 Quintas<br>Natal-RN 59040-000<br>Brazil
	Corporación Científica Pediátrica-Comité de Ética en Investigación Biomédica<br>Calle 5 B5 N0 37 Bis-28<br>Cali-Valle del Cauca 760021<br>Colombia
	Comité de Bioética de Investigación del Hospital Maternidad<br>Nuestra Señora de la Altagracia

	Calle Pedro Henríquez Ureña #49<br>Santo Domingo<br>República Dominicana
	Comité de Ética para Investigaciones Biomédicas (CEIB)<br>Universidad Nacional Autónoma de Nicaragua<br>UNAN, Campo Médico<br>León<br>Nicaragua
	Comité de Bioética en Investigación del Hospital del Niño<br>Panamá, Ave. Balboa, Calle 34<br>0816-00383 Panamá<br>República De Panamá
	University of the Philippines Manila Research Ethics Board<br>2/F Paz Mendoza, 547 Pedro Gil St., Ermita<br>Manila, 1000<br>Philippines
	Research Institute for Tropical Medicine Institutional Review Board<br>Filinvest Corporate City, Alabang<br>Muntinlupa, 1781<br>Philippines
	Independent Ethics Committee De La Salle Health Science Institute<br>Ground Floor De La Salle Angelo King Medical Research Center<br>Congressional Avenue<br>Dasmarinas, Cavite, 4114<br>Philippines
	Advarra Institutional Review Board<br>6940 Columbia Gateway Drive, Suite 110, Columbia, MD, USA<br>21046
	Walter Reed Army Institute of Research Institutional Review Board<br>503 Robert Grant Avenue, Silver Spring, MD, USA<br>20910-7500
	Chong Hua Institutional Review Board<br>Chong Hua Hospital<br>Don Mariano Cui Street, Fuente Osmeña<br>Cebu City, 6000<br>Philippines
	Ethical Review Committee for Research in Human Subjects<br>Ministry of Public Health<br>The Office of the Secretary, Department of Medical Services<br>3rd Floor Of the building No.2, Ministry of Public Health

	Muang, Nonthaburi, 11000<br>Thailand
	Ethics Committee of the Faculty of Tropical Medicine, Mahidol University<br>4th floor, the 60th Ann. Of King´s Accession to the Throne Building<br>Faculty of Tropical Medicine, Mahidol University<br>Bangkok, 10400<br>Thailand
	Ethics Committee of Buddhasothorn hospital<br>174 Marupong Road, Muang District<br>Chachoengsao, 24000<br>Thailand
	Office of the Khon Kaen Ethics Committee in Human Research<br>Academic and Research Laboratory Building (Wechwichakarn),<br>3rd Floor Room 5317 Faculty of Medicine Khon Kaen University<br>Khon Kaen, 40002<br>Thailand
	The Institutional Review Board, Royal Thai Army Medical Department<br>Pharmongkutklao College of Medicine, 317 Rajavithi Road, Rajathevee<br>Bangkok, 10400<br>Thailand
