# Supplementary material for: Bridging the immunogenicity of a tetravalent dengue vaccine (TAK-003) from children and adolescents to adults
Source: NPJ Vaccines. 2023 May 25;8:75. doi: 10.1038/s41541-023-00670-6 (PMC10208910; doi:10.1038/s41541-023-00670-6)
Supplement: Supplementary file 2 — REPORTING SUMMARY [file 41541_2023_670_MOESM2_ESM.pdf]

nature portfolio

Corresponding author(s): Shibadas Biswal

Last updated by author(s): Jan 23, 2023

Reporting Summary

Nature Portfolio wishes to improve the reproducibility of the work that we publish. This form provides structure for consistency and transparency in reporting. For further information on Nature Portfolio policies, see our Editorial Policies and the Editorial Policy Checklist.

Statistics

For all statistical analyses, confirm that the following items are present in the figure legend, table legend, main text, or Methods section.

n/a	Confirmed	
☐	☒	The exact sample size (*n*) for each experimental group/condition, given as a discrete number and unit of measurement
☒	☐	A statement on whether measurements were taken from distinct samples or whether the same sample was measured repeatedly
☐	☒	The statistical test(s) used AND whether they are one- or two-sided<br>*Only common tests should be described solely by name; describe more complex techniques in the Methods section.*
☐	☒	A description of all covariates tested
☐	☒	A description of any assumptions or corrections, such as tests of normality and adjustment for multiple comparisons
☐	☒	A full description of the statistical parameters including central tendency (e.g. means) or other basic estimates (e.g. regression coefficient) AND variation (e.g. standard deviation) or associated estimates of uncertainty (e.g. confidence intervals)
☒	☐	For null hypothesis testing, the test statistic (e.g. *F*, *t*, *r*) with confidence intervals, effect sizes, degrees of freedom and *P* value noted<br>*Give P values as exact values whenever suitable.*
☒	☐	For Bayesian analysis, information on the choice of priors and Markov chain Monte Carlo settings
☒	☐	For hierarchical and complex designs, identification of the appropriate level for tests and full reporting of outcomes
☒	☐	Estimates of effect sizes (e.g. Cohen's *d*, Pearson's *r*), indicating how they were calculated

Our web collection on statistics for biologists contains articles on many of the points above.

Software and code

Policy information about availability of computer code

Data collection	No software was used for data collection.
Data analysis	All statistical analysis was performed using SAS version 9.2 or higher.

For manuscripts utilizing custom algorithms or software that are central to the research but not yet described in published literature, software must be made available to editors and reviewers. We strongly encourage code deposition in a community repository (e.g. GitHub). See the Nature Portfolio guidelines for submitting code & software for further information.

Data

Policy information about availability of data

All manuscripts must include a data availability statement. This statement should provide the following information, where applicable:

- Accession codes, unique identifiers, or web links for publicly available datasets
- A description of any restrictions on data availability
- For clinical datasets or third party data, please ensure that the statement adheres to our policy

The datasets, including the redacted study protocol, redacted statistical analysis plan, and individual participants data supporting the results of the completed studies, will be made available within three months from initial request, to researchers who provide a methodologically sound proposal. The data will be provided after its de-identification, in compliance with applicable privacy laws, data protection and requirements for consent and anonymization. Data requests should follow the process described in the Data Sharing section on https://clinicaltrials.takeda.com/ and https://vivli.org/ourmember/takeda/.

Human research participants

Policy information about studies involving human research participants and Sex and Gender in Research.

Reporting on sex and gender	DEN-301: Sex/gender-based analyses were conducted in the DEN-301 trial.<br><br>DEN-304: There have been no sex/gender-based analyses in the DEN-304 trial. These analyses were not part of the study protocol-defined endpoints, nor were done in an exploratory / post-hoc manner. Of note, the demographic and baseline characteristics were similar across the four study groups (participants have been randomly assigned to one of these four groups - this is the gold standard methodology to obtain well-balanced study groups in terms of demographics). There was also a fairly well-balanced representation of male and female participants (in the PPS, there were 45.9% of male participants and 54.1% of female participants). As previously mentioned, only a subset of patients from this trial were included in the submitted manuscript.
Population characteristics	DEN-301: The mean age of participants from the DEN-301 study was 8.5 years, 49.6% were male, and the mean body mass index (BMI) was 17.3 kg/m2. The racial groups the greatest representation were American Indian or Alaska Native and Asian, with 42.2 and 42.7% respectively.<br><br>DEN-304: Participants from the DEN-304 study had a mean age of 41.2 years, 46.7% were male, and the mean body mass index (BMI) was 27.9 kg/m2. Most participants were white (78.9%) with a minority of Hispanic or Latino (6.1%) ethnicity.
Recruitment	DEN-301: Eligible participants were 4–16 year-old, living in regions of Asia and Latin America considered endemic for dengue, and in good health at the time of enrollment as determined by medical history, physical examination (including vital signs) and the clinical judgment of the investigator, who were able to comply with trial procedures and available for the duration of follow-up. Participants and/or the participant's parent or guardian were required to sign and date a written informed consent form and any required privacy authorization prior to the initiation of any trial procedures, after the nature of the trial had been explained according to local regulatory requirements.<br><br>DEN-304: Eligible participants were 18–60 year-old adults, living in parts of the United States considered non-endemic for dengue, and in good health at the time of enrollment as determined by medical history, physical examination (including vital signs) and the clinical judgment of the investigator, who were able to comply with trial procedures and available for the duration of follow-up. Participants were required to sign and date a written informed consent form and any required privacy authorization prior to the initiation of any trial procedures, after the nature of the trial had been explained according to local regulatory requirements.
Ethics oversight	DEN-301: The trial was conducted in accordance with the Declaration of Helsinki and the International Council for Harmonisation Tripartite Guidelines for Good Clinical Practice, as well as in accordance with applicable local regulations. Informed assent or consent forms and the trial protocol and its amendments were reviewed and approved by institutional review boards, independent ethics committees, and health authorities. Written informed assent or consent was obtained from all participants or their parents or legal guardians before enrollment. During the trial, consent was obtained again from participants when they legally became adult.<br><br>DEN-304: The trial was conducted in accordance with the Declaration of Helsinki and the International Council for Harmonisation Tripartite Guidelines for Good Clinical Practice, as well as in accordance with applicable local regulations. Informed assent or consent forms and the trial protocol and its amendments were reviewed and approved by institutional review boards, independent ethics committees, and health authorities. Written informed assent or consent was obtained from all participants before enrollment.

Note that full information on the approval of the study protocol must also be provided in the manuscript.

Field-specific reporting

Please select the one below that is the best fit for your research. If you are not sure, read the appropriate sections before making your selection.

☒ Life sciences ☐ Behavioural & social sciences ☐ Ecological, evolutionary & environmental sciences

For a reference copy of the document with all sections, see nature.com/documents/nr-reporting-summary-flat.pdf

Life sciences study design

All studies must disclose on these points even when the disclosure is negative.

Sample size	To minimize the potential for confounding factors, we restricted the analysis to participants who were dengue seronegative at baseline (i.e., reciprocal titer of dengue-neutralizing antibodies < 10 for all four serotypes).<br><br>702 baseline seronegative children and adolescents from the DEN-301 trial and 379 baseline seronegative adults from the DEN-304 trial were

	included in this analysis.
Data exclusions	Exclusion criteria for both DEN 301 and DEN-304 trials were predefined and are reported on clinical trial.gov webpages for NCT02747927 and NCT03423173 respectively. Participants from both trials who were baseline seronegative were selected for inclusion in the submitted immunobridging study.
Replication	Geometric mean titers (GMTs) were provided with 95% CIs (figures 1, 3 and 4). As previously described by Rivera et al, 2021(cited in submitted manuscript), the highest GMTs in both age groups were observed against DENV-2, with lower GMTs against the other three serotypes.
Randomization	DEN-301: Children and adolescents 4–16 years of age who met the trial entry criteria were randomized using an interactive Response Technology (IRT) 2:1 ratio to receive two doses of vaccine or placebo, 3 months apart. Randomization was stratified according to region (Asia-Pacific region or Latin America) and age (4–5 years, 6–11 years, or 12–16 years).<br><br>DEN-304: Participants aged 18–60 were randomized using an interactive Response Technology (IRT) 1:1:1:1 to receive one of three lots of the two-dose TAK-003 formulation, or placebo, administered 3 months apart.<br><br>The above randomization criteria relates to the individual DEN-301 and DEN-304 trials, 702 baseline seronegative children and adolescents from the DEN-301 trial and 379 baseline seronegative adults from the DEN-304 trial were included in the submitted immunobridging study.
Blinding	DEN-301: This was a double-blind trial. During the trial, investigators, participants and their parents or guardians, and representatives of the sponsor who advise on trial conduct remain unaware of the trial-group assignments. One or more designated pharmacists or vaccine administrators at each site are aware of the trial-group assignments but have no role in the collection or assessment of participant safety data.<br><br>DEN-304: This was a double-blind trial. During the trial, investigators, participants and representatives of the sponsor who advise on trial conduct remain unaware of the trial-group assignments. One or more designated pharmacists or vaccine administrators at each site are aware of the trial-group assignments but have no role in the collection or assessment of participant safety data.

Reporting for specific materials, systems and methods

We require information from authors about some types of materials, experimental systems and methods used in many studies. Here, indicate whether each material, system or method listed is relevant to your study. If you are not sure if a list item applies to your research, read the appropriate section before selecting a response.

Materials & experimental systems

n/a	Involved in the study
☐	☒ Antibodies
☒	☐ Eukaryotic cell lines
☒	☐ Palaeontology and archaeology
☒	☐ Animals and other organisms
☐	☒ Clinical data
☒	☐ Dual use research of concern

Methods

n/a	Involved in the study
☒	☐ ChIP-seq
☒	☐ Flow cytometry
☒	☐ MRI-based neuroimaging

Antibodies

Antibodies used	Antibodies were not purchased for this study. The antibodies produced were from vaccine-induced immune responses (from participants).
Validation	Antibodies were not validated.

Clinical data

Policy information about clinical studies

All manuscripts should comply with the ICMJE guidelines for publication of clinical research and a completed CONSORT checklist must be included with all submissions.

Clinical trial registration	NCT02747927 and NCT03423173 for DEN-301 and DEN-304 respectively
Study protocol	Clinical trials.gov webpages for the above identifiers
Data collection	DEN-301 and DEN-304: comparisons of GMTs and seropositivity between participants in the two studies were performed at months 4 and 9 (i.e., one and six months after receipt of the second dose of TAK-003).<br><br>DEN-304 only: we characterized the spectrum of immune responses using samples from DEN-304 collected on month 0, 4 and 9 from 48 baseline seronegative participants.

Outcomes

Immunogenicity measures
Immunogenicity was assessed in terms of GMTs of dengue-neutralizing antibodies, as measured by a dengue microneutralization titer assay resulting in ≥ 50% reduction in titer (MNT50).

Exploratory immunology analysis
Exploratory immunology analyses included the following outcomes: Geometric mean concentrations of anti-DENV NS1, Antibody avidity, Anti-dengue complement-fixing antibody titers, and Dengue total binding IgG concentrations. Geometric mean concentrations of anti-DENV NS1 were evaluated using the methods previously described in full in Sharma et al., 2020. Antibody avidity was measured using Octet RED or HTX systems, as described in Tsuji et al., 2021. Anti-dengue complement-fixing antibody titers were assessed using the Luminex assay, as described in full in Nascimento et al., 2021. Dengue total binding IgG concentrations were assessed as described in Michlmayr et al., 2021. Type-specific neutralizing antibody responses against DENV-1, -3, and -4 were evaluated using DENV-2 depletion and RVP assay, as described in DeMaso et al. 2022. All aforementioned references were cited in the submitted manuscript.
